# Buprenorphine as a Treatment for Major Depression and Opioid Use Disorder

**DOI:** 10.3389/adar.2022.10254

**Published:** 2022-02-21

**Authors:** Amanda B. Namchuk, Irwin Lucki, Caroline A. Browne

**Affiliations:** ^1^ Department of Pharmacology and Molecular Therapeutics, Uniformed Services University, Bethesda, MD, United States; ^2^ Department of Psychiatry, Uniformed Services University, Bethesda, MD, United States

**Keywords:** buprenorphine, opioid use disorder, suicide, major depressive disorder, methadone

## Abstract

Rates of major depressive disorder (MDD) are disproportionally high in subjects with opioid use disorder (OUD) relative to the general population. MDD is often more severe in OUD patients, leading to compliance issues with maintenance therapies and poor outcomes. A growing body of literature suggests that endogenous opioid system dysregulation may play a role in the emergence of MDD. Buprenorphine, a mixed opioid receptor agonist/antagonist approved for the treatment of OUD and chronic pain, may have potential as a novel therapeutic for MDD, especially for patients with a dual diagnosis of MDD and OUD. This paper presents a comprehensive review of papers relevant to the assessment of buprenorphine as a treatment for MDD, OUD, and/or suicide compiled using electronic databases per Preferred Reporting Items for Systematic Reviews and Meta-Analyses (PRISMA) guidelines. The principal goal of this literature review was to compile the clinical studies that have interrogated the antidepressant activity of buprenorphine in opioid-naïve MDD patients and OUD patients with comorbid MDD. Evidence supporting buprenorphine’s superiority over methadone for treating comorbid OUD and MDD was also considered. Finally, recent evidence for the ability of buprenorphine to alleviate suicidal ideation in both opioid-naïve patients and opioid-experienced patients was evaluated. Synthesizing all of this information, buprenorphine emerges as a potentially effective therapeutic for the dual purposes of treating MDD and OUD.

## Introduction

In the late 1990s, pharmaceutical companies began marketing opioid analgesics to physicians as not only safe and non-addictive, but essential for quality patient care. Pain joined temperature, blood pressure, respiratory rate, and heart rate as “the fifth vital sign” as doctors started to consistently prescribe opioid analgesics for acute and chronic pain (1). The consequent rise in prescription opioid use has been linked directly to an initial increase of overdose deaths starting in 1999 (2). By 2010, it became clear that opioid analgesics were, in fact, “addictive” and efforts were made to reduce opioid prescribing. As many opioid-dependent patients were forced to seek alternative nonprescription methods to access opioids, the rate of heroin use increased leading to a resurgence in deaths from opioid overdose (3). In 2013, dealers began to cut heroin and other drugs with the potent synthetic opioid fentanyl and deaths from opioid overdose spiked yet again (4). Approximately three million US citizens have suffered or are currently suffering from opioid use disorder (OUD) (5), spurred largely by the overprescribing of opioid analgesics that were originally mischaracterized as non-addictive. The ongoing opioid crisis in the United States has created an enormous public health and economic burden, causing 128 overdose deaths every day (6) and costing $78.5 billion a year from medical care and treatment, lost productivity, and criminal justice activity (7).

In order to stymie the epidemic, it is imperative to appropriately treat each patient’s OUD, taper them off medication, and reintegrate them into society. Unfortunately, this process is often complicated by difficult social and economic circumstances, polydrug use, and extensive psychiatric comorbidity. This review focuses specifically on the large population of OUD patients with comorbid major depressive disorder (MDD). Individuals with OUD are significantly more likely to be depressed than the general population (8). These individuals are harder to treat because they are less likely to achieve long-term OUD remission (9) and are at greater risk for unintentional and intentional suicide attempts (10).

To this end, buprenorphine has emerged as an intriguing pharmacotherapeutic candidate for both OUD and MDD. Buprenorphine is a semi-synthetic opioid that acts as a partial mu-opioid receptor (MOR) agonist and an antagonist at the kappa opioid receptor (KOR) and delta opioid receptor (DOR). It is approved by the Food and Drug Admiration (FDA) for the treatment of OUD and chronic pain, but not depression (11). Although there is anecdotal evidence throughout history of the antidepressant effects of opium and other substances derived from the poppy plant, opioids were largely abandoned for the treatment of depression as monoamine-based antidepressants were introduced (12). Unfortunately, modern antidepressant therapeutics are not very effective because less than 50% of patients with depression respond to therapy with first-line antidepressants (13). Approximately 30–40% of MDD patients have treatment-resistant depression (TRD), meaning they have failed to respond to adequate trials of two or more antidepressant medications (14,15). Thus, there is a considerable unmet need for new treatment options for the TRD population with and without comorbid OUD.

Due to recent advances in understanding opioid pharmacology, opioid system modulation has emerged as a potential target for novel antidepressant development (15). While the mechanisms underlying opioid system modulation for the treatment of depression have yet to be fully elucidated, clinical studies of buprenorphine and related opioid drugs show promise for producing clinically significant effects. The goal of this review is to comprehensively consider the clinical evidence for the antidepressant effects of buprenorphine in different populations—opioid-naïve MDD patients, OUD patients with MDD, suicidal opioid-naïve patients, and suicidal opioid-experienced patients. Furthermore, because buprenorphine and methadone are the leading treatments for OUD, the antidepressant effects of methadone and buprenorphine for OUD patients will be compared with and without a clinical MDD diagnosis.

## Methods

**FIGURE 1:**
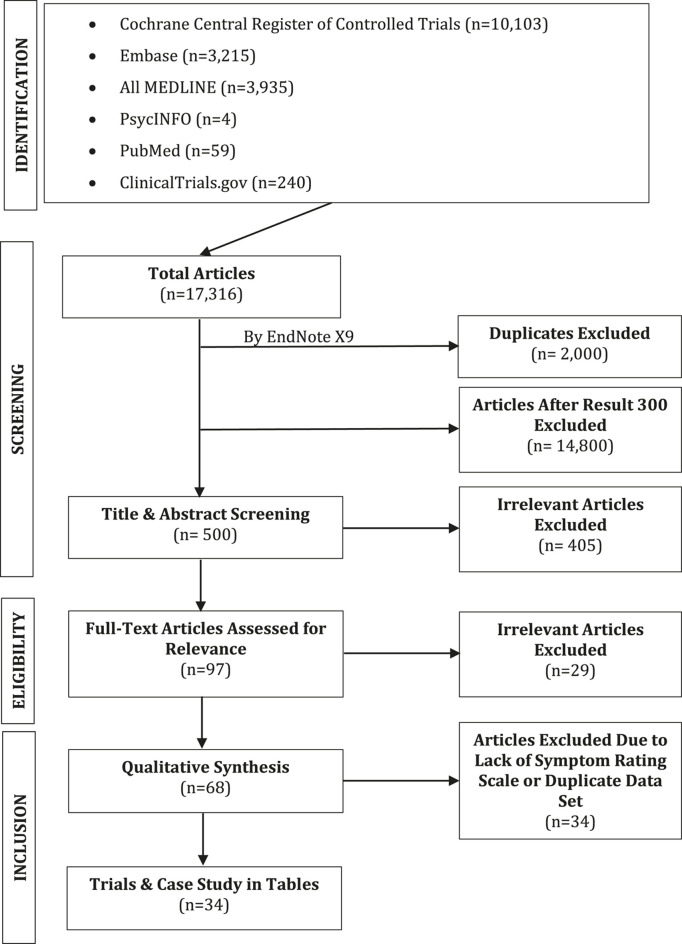
PRISMA flow diagram of studies’ screening and selection.

In line with PRISMA (16) a review of the relevant literature was conducted as outlined in Figure 1. Briefly, a power search was conducted through the USUHS online library, powered by Ex Libris Alma/Primo, comprising the library catalog, articles, and EBSCO databases. This facilitated the search of Cochrane Central Register of Controlled Trials, CINAHL, Embase, MEDLINE, MEDLINE In-Process, PsycINFO, and NCBI PubMed from the date of their inception to 7 January 2022, with no language restrictions. The search terms were “buprenorphine” AND “substance use disorder” AND “major depressive disorder.” The electronic database searches for relevant clinical studies/trials were supplemented with manual searches for published, unpublished, and ongoing trials in ClinicalTrials.gov using the search terms “major depressive disorder” or “substance use disorder” combined with “buprenorphine.”

Due to the heterogenous nature of the data set and the lack of placebo-controlled trials for buprenorphine alone, meta-analyses were not performed. As such a comprehensive evaluation on all the relevant literature was conducted.

## Low-Dose Buprenorphine Acts as a Rapid Antidepressant in the Opioid-Naïve Population

### Open Trials

The first evidence that low doses of buprenorphine could be used for treating depression in patients without a history of OUD appeared in a series of published open trials (Table 1), starting in 1982 (17). Using a double-blind crossover design and .2 mg/day of sublingual buprenorphine, it was found that among 10 depressed patients, most of whom were treatment resistant, half experienced a significant improvement in depressive symptoms. Despite half of the patients being non-responders, the average reduction in Hamilton Rating Scale for Depression (HAM-D) score was 40% (18), falling from 27 on average to approximately 14 at peak efficacy (17). The rate of remission was not reported. As .2 mg/day is a very low dose, it is possible that more patients would have responded had they received a higher fixed dose or if flexible dosing of buprenorphine had been allowed. Since the publication of this provocative positive result, many other researchers (Table 1) have assessed the antidepressant effects of buprenorphine both alone (19–23) and in combination with samidorphan, a MOR antagonist (24, 25).

**TABLE 1:** Trials of Buprenorphine in the Opioid-Naïve Population. Low-dose buprenorphine in opioid-naïve patients consistently ameliorates depressive symptoms both alone and in combination with samidorphan—and does so quickly.

References	Study design	Subjects	Dose: mean (SD)	MDD rating scale	Baseline score: mean (SD)	Final score: mean (SD)	Outcome
(17,18)	Open trial, crossover design	10 MDD patients (most TRD); no control	.2 mg/day (fixed)	HAM-D	∼27	∼14	50% responded; average HAM-D score reduction was 40%
(19)	Open trial 4–6 weeks	10 TRD patients (7 completed trial); no control	1.26 (.55) mg/day	HAM-D	28.1 (6.6)	10.7 (9.3)	Significant reduction in HAM-D within 1 week; overall HAM-D score. Reduction of 60.7%; 4/7 subjects achieved remission
(20)	Open trial	3 TRD, 1 dysthymia, 1 chronic fatigue syndrome + pain + dysphoria; no control	Not reported	N/A	N/A	N/A	All 5 patients responded to BUP and results were maintained “several years” later
(23)	Open trial 1 week	6 TRD patients; no control	1.2 (.4) mg/day	HAM-D	22.8 (4.5)	6.0 (3.2)	All 6 patients had a significant antidepressant response; 5 achieved remission by the HAM-D and 4 b y the BDI.
				BDI	34.3 (12.5)	12.8 (7.9)	
(21) (NCT01071538)	Open trial 8 weeks	15 TRD patients, 50+ years old (13 completed trial); no control	.4 (.21) mg/day	MADRS	27.0 (7.3)	9.5 (9.5)	Augmentation study (2 patients used BUP at monotherapy); mean score change in first week was -15.0 (SD = 7.9); 66.7% had a response (MADRS ≤10) at any timepoint; 61.5% maintained response at 8 weeks
(22) (NCT02176291)	RCT (Phase 2) 8 weeks	30 TRD patients, 50+ years old: 16 BUP and 11 placebo included in analysis	.5 (.2) mg/day	MADRS	24 (6)	18 (9)	Augmentation study: all participants experienced improvement in depressive symptoms but no difference in final MADRS score or % MADRS score change between BUP and placebo groups
			Placebo		27 (5)	15 (9)	
(24) (NCT01381107)	RCT (Study 2) 1 week	18 TRD patients: 14 BUP/SAM treated and 4 placebo	4 mg/4 mg BUP/SAM--> 8 mg/8 mg BIP/SAM/day	HAM-D	19.4 (2.7)	12.7 (3.4)	Augmentation study; 1:1 BUP/SAM group experienced a statistically significant improvement on the HAM-D, and an effect approaching significance on the MADRS.
			Placebo		19.0 (3.2)	18 (4.2)	
			4 mg/4 mg BUP/SAM--> 8 mg/8 mg BUP/SAM/day	MADRS	26.4 (4.4)	14.9 (6.5)	
			Placebo		24.5 (7.9)	21 (5.8)	
(25) (NCT01500200)	RCT, SPCD	135 TRD patients: 20 2 mg/2 mg BUP/SAM treated, 20 8 mg/8 mg BUP/SAM treated, 95 placebo	2 mg/2 mg BUP/SAM/day	HAM-D	22.7 (4.2)	13.0 (6.3)	Augmentation study: 2/2 group had significant reduction in depressive symptoms compared to placebo as measured by the HAM-D, MADRS, and CGI-S; reduction in depressive symptoms in 8/8 group compared to placebo did not reach significance on any measure
			8 mg/8 mg BUP/SAM/day		21.7 (3.3)	15.4 (6.6)	
			Placebo		23.2 (4.2)	15.8 (7.3)	
			2 mg/2 mg BUP/SAM/day	MADRS	31.3 (5.6)	17.0 (10.4)	
			8 mg/8 mg BUP/SAM/day		28.1 (4.1)	17.6 (11.3)	
			Placebo		31.0 (5.6)	21.2 (10.7)	
	Stage 1: 4 weeks		2 mg/2 mg BUP/SAM/day	CGI-S	4.5 (.5)	3.1 (1.0)	
			8 mg/8 mg BUP/SAM/day		4.6 (.6)	3.3 (1.1)	
			Placebo		4.4 (.5)	3.5 (1.2)	
		65 TRD patients (non-responders from Stage 1 placebo group): 23 2 mg/2 mg BUP/SAM treated, 22 8 mg/8 mg BUP/SAM treated, 20 placebo	2 mg/2 mg BUP/SAM/day	HAM-D	16.1 (5.9)	11.1 (7.3)	
			8 mg/8 mg BUP/SAM/day		19.0 (5.5)	15.1 (6.6)	
			Placebo		17.3 (8.8)	15.9 (7.8)	
	Stage 2: 4 weeks		2 mg/2 mg BUP/SAM/day	MADRS	21.6 (9.0)	12.5 (10.4)	
			8 mg/8 mg BUP/SAM/day		26.2 (7.4)	20.2 (10.1)	
			Placebo		23.6 (12.5)	21.5 (11.0)	
			2 mg/2 mg BUP/SAM/day	CGI-S	3.5 (1.1)	2.4 (1.2)	
			8 mg/8 mg BUP/SAM/day		4.2 (.8)	3.3 (1.0)	
			Placebo		3.6 (1.2)	3.2 (1.2)	

MDD, major depressive disorder; TRD, treatment-resistant depression; BUP, buprenorphine; SAM, samidorphan; RCT, randomized controlled trial; SPCD, sequential parallel comparison design; HAM-D, hamilton rating scale for depression; BDI, beck depression inventory; MADRS, Montgomery-Åsberg Depression Rating Scale; CGI-S, clinical global impressions severity scale; SD, standard deviation.

In a following report, 10 TRD patients were treated with an average dose of 1.26 mg/day of buprenorphine (maximum dose: 1.8 mg/day), seven of whom completed 4 weeks of treatment and four of whom completed 6 weeks (19). At the endpoint (four or 6 weeks depending on the subject), the average HAM-D score declined from 28.1 to 10.7, a 60.7% reduction in rating of depressive symptoms. Furthermore, four subjects achieved remission by scoring six or less on the HAM-D. This outcome was notable for a group that had previously failed to respond to an average of 7.6 antidepressants. Similarly, in 1996, Callaway (19, 20) reported long-term success in alleviating the symptoms of three patients diagnosed with treatment resistant depression, one with dysthymia and one with chronic fatigue, pain and dysphoria. Unfortunately, the specific methods of the study were not reported.

In a 1-week open trial, Nyhuis et al. (23), treated six longtime TRD inpatients with .8–2.0 mg/day of buprenorphine. All six patients experienced a significant improvement in depressive symptoms, with the average HAM-D rating falling from 22.8 to 6.0 and the average Beck Depression Inventory (BDI) score decreasing from 34.3 to 12.8 after 7 days of treatment. Five patients achieved remission, an impressive outcome considering that these inpatients had consistently failed to respond to multiple antidepressants given for months prior to the study.

Karp et al. (21), reported positive results from an 8-week open label trial of buprenorphine for TRD in adults 50 years or older. The treatment regimen of 13 participants was augmented with buprenorphine while two other subjects utilized buprenorphine as a monotherapy. Using an average dose of .4 mg/day (range: .12–.83 mg/day), the mean Montgomery-Åsberg Depression Rating Scale (MADRS) score dropped from 27.0 to 9.5. With a significant response defined as a MADRS score ≤10, eight out of 13 participants achieved this remission at the 8-week timepoint.

### Randomized Controlled Trials

Building on the results of their first open study, Karp et al. conducted a follow-up RCT (completed in 2018) investigating TRD treatment augmentation with buprenorphine in adults over 50. Some data from the University of Pittsburgh trial site have been published (22), but it appears the group was unable to replicate their previous findings. This trial had two phases: Phase 1 was a 12-week open treatment period where all patients received up to 300 mg/day of venlafaxine XR; patients with a MADRS score >10 for at least 2 weeks at the end of Phase 1 were verified “non-responders,” and randomized into the buprenorphine or placebo group for eight additional weeks of treatment as an adjunct therapy. The analysis included 16 buprenorphine-treated subjects and 11 placebo-treated subjects with a final mean buprenorphine dose of .5 mg/day. At the beginning of Phase 2, the buprenorphine group average MADRS score was 24 and the placebo group score was 27. After 8 weeks, the buprenorphine group MADRS score had fallen to 18 and the placebo group score was 15. The antidepressant effect of buprenorphine was no greater than that of placebo, although this result may have been driven by a large placebo effect (MADRS score changed from 27 to 15) rather than inefficacy of buprenorphine. This paper was primarily concerned with neuroimaging correlates, so future publications from the same trial may explore the behavioral effects of buprenorphine further.

Buprenorphine has also been studied in combination with samidorphan, an MOR antagonist developed by Alkermes, Inc. Ehrich et al. (24) conducted a 7 day parallel-group randomized controlled trial of a 1:1 ratio of buprenorphine and samidorphan (4 mg/4 mg on days one and two; 8 mg/8 mg on days three to seven) in MDD patients who were taking a standard antidepressant but had failed to achieve a 50% reduction in symptoms. With the addition of the adjunctive therapy for 1 week, the 14 treated patients exhibited a statistically significant decrease in HAM-D scores (score change of −6.7) compared to that of four patients receiving placebo. The MADRS score change of −11.5 in the active treatment arm approached statistical significance.

The results of an expanded multi-site follow-up study further demonstrated the utility of a 1:1 combination of buprenorphine and samidorphan as an adjunctive therapy for TRD (25). This Sequential Parallel Comparison Design (SPCD) study had two stages. Stage 1 split participants into a 2 mg/2 mg buprenorphine/samidorphan treatment group, an 8 mg/8 mg buprenorphine/samidorphan treatment group, and a placebo group. Stage 2 removed placebo responders and re-randomized the non-responders. Both stages included a 4-week treatment period and a 1-week taper. In Stage 1, 20 patients were randomized to the 2/2 treatment group and 20 to the 8/8 treatment group. Subjects who received buprenorphine/samidorphan in Stage 1 received placebo for the duration of Stage 2. Of the 95 patients that received placebo in Stage 1, 20 were randomized to the placebo group for Stage 2, 23 were randomized to the 2/2 treatment group, and 22 to the 8/8 treatment group. See Table 1 for baseline and 4-week HAM-D, MADRS, and Clinical Global Impressions severity scale (CGI-S) raw scores.

A treatment response was defined as a ≥50% reduction in depressive symptoms as measured by the HAM-D and/or the MADRS. As measured by the HAM-D, at the 4-week timepoint 47% of patients had responded to the 2/2 combination in Phase 1 and 33% had responded in Phase 2. The rate of response to the 2/2 combination as measured by the MADRS was 41% in Phase 1 and 50% in Phase 2. Rates of remission as measured by both the HAM-D and MADRS were similar to response rates. With both phases analyzed together, at the 4-week timepoint the 2/2 group exhibited significant improvements compared to baseline on the HAM-D (placebo-adjusted least-squares mean difference of −2.8), MADRS (placebo-adjusted least-squares mean difference of −4.9), and CGI-S (placebo-adjusted least-squares mean difference of −.5). The 2/2 group outperformed the 8/8 group whose HAM-D, MADRS, and CGI-S mean score changes failed to reach significance, possibly due to adverse side effects elicited at a higher dose (25). Regardless, this study presented further evidence that a low dose of buprenorphine can effectively relieve depressive symptoms.

### Buprenorphine Abuse Liability in an Opioid-Naïve Population

Prescribing buprenorphine off-label in non-opioid experienced patients with MDD has raised a broader concern regarding the potential abuse liability and diversion of buprenorphine. The issue requires balancing the medical value of developing a novel antidepressant for otherwise treatment-resistant patients versus the burden that could be caused by potential abuse-related effects.

It is important to note that many animal and human studies have shown that buprenorphine, as a partial mu receptor agonist, is inherently less rewarding and shows much less abuse potential (11, 26) and cognitively impairing effects relative to other full MOR agonists (27). Although there is a clear potential for abuse liability in non-dependent individuals with a history of heroin use for buprenorphine alone, the combination of buprenorphine/naloxone significantly mitigates this risk (28–30) as does the use of extended release buprenorphine depots (31). In addition, the doses used in the antidepressant trials (.2–.5 mg) were at least 10-fold lower than the range used to treat OUD and, in most patients, produced minimal side effects (19–25). Moreover, none of the buprenorphine trials reported clinically significant withdrawal symptoms upon buprenorphine discontinuation (21, 24, 25).

Doses of buprenorphine in this range do not increase subjective ratings of euphoria relative to placebo treated individuals in opioid naïve healthy controls using the Drug Effects Questionnaire DEQ (32). Moreover, the effects of a single administration of .2 mg of sublingual buprenorphine to normal volunteers has been shown to dampen negative responses to psychosocial stimuli such as public speaking (33, 34), social rejection (32), emotional faces (32, 35, 36), and images with social content (32,35) and to improve memory of social reward (37). Additional studies should be conducted to examine MDD patients for similar behavioral effects including the DEQ, to determine whether a diagnosis of MDD impacts the subjective ratings of buprenorphine’s effects. Without reported evidence, it is possible that doses of buprenorphine in this range may not have significant abuse liability in this patient population.

Some opioid-naïve patients may discontinue taking buprenorphine after experiencing acute opioid side effects. A small number of dropouts was common in the studies, with rates ranging from 0 to 31.6% (Stage 1 8/8 dosage group, Fava et al. (25)). While the side effects of buprenorphine may be too much for some, low doses of buprenorphine are generally well-tolerated by opioid-naïve patients.

The use of the buprenorphine/samidorphan combination mentioned above addressed the concern of potential abuse by using a mu receptor antagonist (samidorphan) to block any abuse-related effects of buprenorphine. The goal of minimizing the potential abuse liability associated with buprenorphine while maintaining its antidepressant efficacy is likely to favor the use of such drug combinations, such as buprenorphine/naloxone formulations, going forward. Furthermore, the use of injectable extended-release buprenorphine, which has a 43-to-60-day half-life, would be expected to mitigate diversion of the drug, although its long-term antidepressant effects have not yet been evaluated.

### Section Summary

Compared to traditional monoamine-based antidepressants which generally take about 6 weeks to produce behavioral effects, buprenorphine appears to work quickly. With the exception of two papers (22, 25), all studies cited demonstrated antidepressant efficacy in patients with TRD within just 1 week of treatment (19–21, 23, 24). In the case of the two studies published by Karp et al., the authors note that MDD can present with a slightly different symptom profile in older adults (21, 22). It is possible that by restricting the subject pool to adults over 50, these trials compared a depressive phenotype that differed from the young adult subject pool.

Taken together, these results from a small number of clinical studies demonstrate that low-dose buprenorphine can rapidly and significantly relieve depressive symptoms in the opioid-naïve population with minimal side effects and safety concerns. These data support the continued investigation of buprenorphine especially for TRD patients when given alone or in combination with samidorphan or other antagonists. The issue of potential abuse liability represents a significant barrier to development despite the clear medical need. Most importantly further research is needed to provide more definitive confirmation of efficacy using randomized, placebo-controlled trials.

## Buprenorphine Can Effectively Treat Major Depressive Disorder With Opioid Use Disorder

Diagnosis of MDD in individuals with OUD occurs more frequently than in the general population. Across studies, the prevalence of lifetime major depression in this group ranges from 38% to 56% and the rate of current major depression ranges from 16% to 30% (8). Depressed OUD patients may be less likely to comply with a buprenorphine treatment regimen and are more likely to drop out of treatment than their non-depressed counterparts (38). Additionally, depressed individuals are more likely to misuse alcohol and other drugs (39) and OUD patients with a co-occurring psychiatric diagnosis are more likely to have an additional drug dependence (40). Indeed, 28% of heroin users entering the Australian Treatment Outcomes Study (ATOS) met criteria for current major depression (41), and those with depression were less likely to achieve long-term OUD remission (9). Interestingly, in the Prescription Opioid Addiction Treatment Study (POATS), a lifetime diagnosis of MDD was the best predictor of a good outcome with buprenorphine treatment for OUD (42). See Ghabrash et al. (43) for a review of the literature investigating the relationship between depression and OUD outcomes. While the direction of causality is unclear and varies from person to person, patients with comorbid MDD and OUD may be more difficult to treat and are most likely to succeed in achieving remission of either disorder when both are adequately treated. Buprenorphine may do just that (Table 2).

**TABLE 2:** Trials of Buprenorphine for MDD with OUD. Buprenorphine at moderate and high doses effectively reduces depressive symptoms in patients with MDD and OUD.

References	Study design	Subjects	Dose: mean (SD)	MDD rating scale	Baseline score: mean (SD)	Final score: mean (SD)	Outcome
(44)	Open trial 4 weeks	21 OUD only patients	3.2 (1.5) mg/day	SDS	6.3 (not reported)	not reported	75% of responders had a significant reduction in SDS within first week; 83% of those who responded did so within 2 weeks; final SDS scores were similar between groups
				BDI	not reported	not reported	
		19 OUD + MDD patients		SDS	9.6 (5.1)	6.5 (4.2)	
				BDI	17.1 (6.4)	not reported	
(45) (NCT01052662)	Open trial 8 weeks	17 OUD patients using heroin	16 mg/day (fixed)	CES-D	23.1 (11.9)	18.58 (10.3)	Only the prescription opioid group experienced a significant reduction in CES-D score
		63 OUD patients using prescription opioids			22.2 (9.4)	9.88 (7.4)	
(51)	RCT 3 days (single administration)	11 OUD + MDD patients	32 mg	BDI	29.00	4.09	All 40 patients achieved remission of depressive symptoms (no between-group differences); effect maintained at 2-week follow up
		14 OUD + MDD patients	64 mg	BDI	27.00	6.42	
		15 OUD + MDD patients	96 mg	BDI	29.73	4.33	
(46)	RCT 12 weeks	24 OUD patients	8.7 (4.1) mg/day	BDI	24.7 (11.0)	13.4 (10.2)	Depressive symptoms improved but group average BDI remained high; see Table 3 for comparison to methadone
(48) (NCT00316277)	RCT 12 weeks	237 OUD patients without lifetime MDD diagnosis	Not reported. Most common doses: 16 and 24 mg/day	BDI	∼21	∼6	Depressive symptoms in both groups were prevalent at baseline and improved significantly in the first 4 weeks; BDI remained higher for the lifetime MDD group
		123 OUD patients with lifetime MDD diagnosis			∼27	∼11	

MDD, major depressive disorder; OUD, opioid use disorder; RCT, randomized controlled trial; SDS, short depression scale; BDI, beck depression inventory; CES-D, center for epidemiologic studies depression scale; SD, standard deviation.

### Open Trials

In the first open trial investigating the antidepressant effects of buprenorphine in OUD patients, Kosten et al. (44) found that sublingual buprenorphine provided rapid-acting and clinically significant antidepressant benefits to depressed patients. The 40 OUD patients entered a 1-month open trial, 19 of whom were classified as depressed as their baseline BDI scores were greater than 10. Even with a relatively low dose averaging 3.2 mg/day (range: 2–8 mg/day), 63% of depressed OUD patients responded to buprenorphine: nine patients had a “good” antidepressant response wherein their Short Depression Scale (SDS) scores decreased by at least 50% and three patients experienced a “fair” antidepressant response with a “less robust” decrease in SDS score (44). Of those who responded, 75% experienced significant improvement within a week and 83% improved within 2 weeks. For the depressed group as a whole, depressive symptoms decreased steadily over the month until the group average SDS score had fallen from 17.1 to 6.5, similar to that of the non-depressed group at the final timepoint (44).

Seeking to differentiate the ability of buprenorphine to treat opioid withdrawal and depressive symptoms in OUD patients who use heroin vs. prescription opioids, Romero-Gonzalez et al. (45) conducted an 8-week open trial of buprenorphine/naloxone. The 63 patients taking prescription opioids and 17 patients using heroin were treated at a fixed dose of 16 mg/4 mg buprenorphine/naloxone. Patients with a current diagnosis of MDD or receiving antidepressant treatment were excluded, although symptoms associated with depression were prevalent at baseline as measured using the Center for Epidemiologic Studies Depression Scale (CES-D). The average baseline CES-D score among the heroin group was 23.1 and decreased insignificantly to 18.58 over 8 weeks, still well above the cutoff of 16 indicating depression. Among the prescription opioid group, the average CES-D score fell from 22.2 at baseline to 9.88 at 8 weeks. In addition to experiencing a significantly greater antidepressant effect, the prescription opioid group also had more favorable OUD outcomes such as retention, compliance, and weekly opioid use. The authors postulate that buprenorphine’s differential efficacy between prescription opioid and heroin users could be because a higher dose was required to elicit the same beneficial effect in heroin users.

### Randomized Controlled Trials

As part of a large study conducted by the National Drug and Alcohol Research Centre in Australia (NDARC), Dean et al. (46) examined depressive symptoms among a cohort of 24 OUD patients on buprenorphine maintenance therapy. All subjects’ depressive symptoms improved significantly over 3 months, with the average BDI score among buprenorphine-treated patients declining from 24.7 to 13.4. The average dose administered was 8.7 mg/day of sublingual buprenorphine (range: 2–32 mg/day), slightly higher compared to Kosten et al., but still quite low for OUD patients (Table 2). No relationship between dose of buprenorphine and final BDI was evident, either because there was a ceiling to buprenorphine’s antidepressant effects, or the lower average dose used in this study masked any potential interaction.

The large, multi-site POATS trial included two phases designed to mimic clinical practice for OUD patients. All participants underwent buprenorphine/naloxone treatment in both phases but were randomized to receive either standard medical management or standard medical management plus individual opioid dependence counseling. Patients who relapsed (610 of 653) after the brief Phase 1 were invited to enter Phase 2 in which they were re-randomized and treated for 12 weeks, followed by a 4-week taper and 8-week follow-up. 360 participants enrolled in Phase 2. Doses of sublingual buprenorphine ranged from eight to 32 mg/day, with the most commonly prescribed doses being 16 and 24 mg/day. At the 12-week timepoint, 49.2% of participants had achieved a “successful outcome,” meaning they had been abstinent for three of the last 4 weeks of the study including week 12. No differences in OUD outcomes were identified in either phase between the standard medical management group and standard medical management plus individual opioid dependence counseling groups (47).

Current psychiatric comorbidity was common among the 360 participants in Phase 2 and turned out to be one of the most important factors in a successful opioid use outcome. 20% of participants had a current MDD diagnosis, 34% had a lifetime MDD diagnosis, and 50% had any current psychiatric diagnosis (42). Participants with a lifetime MDD diagnosis had an average BDI score of about 27 whereas participants who had never had a MDD diagnosis had an average score around 21. For both groups, BDI scores dropped significantly before the next assessment at 4 weeks and stayed relatively steady for the remainder of Phase 2. At week 12 the lifetime MDD group average BDI score was about 11, still significantly higher than that of the non-MDD group which was about 6 (48).

Although OUD patients with a co-occurring psychiatric disorder had greater impairment as indicated by higher scores on the BDI, several domains of the Addiction Severity Index (ASI), and the Short Form-36 (SF-36) which assesses quality of life, these subjects were more likely to have successful opioid use outcomes (40). Indeed, past-year and lifetime diagnoses of MDD were predictors of success. Of the OUD patients who were abstinent at week 12, 46% had an MDD diagnosis in the past year and 73% had a lifetime diagnosis. Among OUD patients who did not achieve abstinence at week 12, 26% had an MDD diagnosis in the past year and 50% had a lifetime MDD diagnosis (42). The positive predictive value of a lifetime diagnosis of MDD could not be explained by a reduction in depressive symptoms due to treatment or greater motivation to engage in treatment. Greater engagement in treatment was associated with better outcomes regardless of lifetime diagnosis of MDD (48). Upon long-term follow-up at 18 and 42 months, past year diagnosis of MDD, lifetime diagnosis of MDD, and higher baseline BDI were no longer predictors of opioid abstinence, although participants with a higher baseline BDI score were more likely to still be engaged in maintenance therapy (49, 50).

High doses of buprenorphine produced rapid and long-lasting effects on depressive scores after just one administration (51). The 40 male psychiatric inpatients diagnosed with both OUD and MDD were randomly assigned a sublingual dose of 32, 64, or 96 mg of buprenorphine, which was administered when the patient was experiencing moderate (four or five out of six) opioid withdrawal symptoms (52). Based on the BDI cutoff of 10, all 40 patients achieved remission of depressive symptoms by day three post-treatment. Average BDI score among the 32 mg group fell from 29.00 to 4.09, 27.00 to 6.42 in the 64 mg group, and 29.73 to 4.33 in the 96 mg group. At the 2-week follow-up, patients continued to exhibit remission from their depressive symptoms (51). There were no between-group differences, further supporting the hypothesis that buprenorphine’s dose-response curve for outcomes related to MDD reaches a plateau.

### Section Summary

Overall, these clinical studies support the use of buprenorphine to treat MDD comorbid with OUD and suggest that further research is warranted. While the randomized nature of several of these trials is a key strength, a significant limitation is the lack of a placebo control group. Ethical and logistical reasons may limit the use of placebo groups in which certain OUD patients receiving no treatment is not a viable option and dose finding or treatment comparison studies may be better options.

It is also important to note that while there is evidence that buprenorphine elicits antidepressant effects at a very wide range of doses (Table 2), interpretability of these data may be limited by the fact that a dose-response curve for buprenorphine has not been established in these patient populations. Regardless, in patients with comorbid MDD and OUD, a higher dose of buprenorphine than is necessary to treat depressive symptoms alone is required to manage opioid craving and withdrawal symptoms. Another difference between dually diagnosed patients and those with depression alone is their sensitivity to the side effects of buprenorphine. As MDD and OUD patients have already been exposed to opioids, they can tolerate higher doses of buprenorphine than opioid-naïve patients. Within the patient population with both MDD and OUD, there may also be a difference in optimal dosage between prescription opioid users and heroin users (45).

Consistent with findings in opioid-naïve patients, data from trials including MDD and OUD subjects support the rapid action of buprenorphine in alleviating depression severity in this patient population compared with conventional antidepressant medications. Nevertheless, randomized controlled trials of buprenorphine for OUD, in which a primary outcome measure is a reduction in depressive symptoms, are lacking. Monitoring the time course of changes to depressive symptoms to differentiate the mood effects of buprenorphine itself from the relief of treating an OUD is a difficult but critical next step in further assessing the utility of buprenorphine for treating MDD in the opioid-experienced population.

## Superiority of Buprenorphine vs. Methadone in Comorbid Major Depressive Disorder/Opioid Use Disorder Remains Unclear

Since the approval of buprenorphine as a pharmacotherapy for OUD, buprenorphine and methadone have been established as the leading treatments for OUD. Although there is variability between studies and conditions of treatment can vary, the two medications seem to be similarly efficacious for the treatment of OUD overall (53), borne out by the data below.

### Open Trials

In a 12-week observational study of 78 longtime OUD patients entering a methadone treatment program and 76 entering a buprenorphine treatment program, Gerra et al. (54) determined that buprenorphine was more effective than methadone for OUD patients with comorbid MDD. The prevalence of comorbid MDD in this cohort was 19.7% among buprenorphine-treated patients and 17.9% among methadone-treated patients. The average dose of methadone was 81.5 mg/day, and the average dose of buprenorphine was 9.2 mg/day. Methadone and buprenorphine-treated patients had similar rates of retention over the 12-week study period (61.5% and 59.2%, respectively), although depressed patients were more likely to be retained for the duration of the study if they were treated with buprenorphine. Moreover, the Symptom Checklist 90 (SCL-90) depression subscale revealed that among patients who completed the 12-week study, buprenorphine-treated patients experienced a more consistent amelioration of depressive symptoms.

There were significant limitations to this study due to its naturalistic design. As the patients chose which treatment program to enroll in, it was not a randomized controlled trial. Therefore, despite seemingly similar demographics, it is possible that differentiating characteristics of patients with OUD and MDD who opted for buprenorphine exist that made them more likely to succeed than those who choose methadone. Nevertheless, buprenorphine was more effective at treating both OUD and MDD regardless of OUD severity and psychosocial functioning.

Another observational study comparing buprenorphine and methadone was conducted by Maremmani et al. (55). This 9-month study was designed to examine outcomes for patients that remained in treatment following the early attrition period, and thus began 3 months after treatment induction. The 106 buprenorphine-treated patients received an average of 7.60 mg/day of the medication at 3 months. At 12 months, the 83 remaining patients received an average of 5.10 mg/day. The 107 methadone-treated patients were given an average dose of 69.4 mg/day at 3 months. The 80 remaining patients were treated with 61.68 mg/day of methadone at 12 months. Although depressive symptoms were relatively mild in this population, the authors reported significant decreases in SCL-90 depression subscale scores between three and 12 months in both groups. Among methadone-treated patients the average score declined from .79 to .67 (15.2%), whereas in the buprenorphine group the average score fell from .60 to .39 (35%). The greater decrease in SCL-90 depression subscale scores among the buprenorphine group suggested that treatment of OUD with buprenorphine was more effective at relieving depressive symptoms than methadone.

It is important to note that one can experience an episode of depressive mood symptoms without meeting both the intensity and duration criteria required for a clinical diagnosis of MDD. This was likely for most patients studied in Maremmani et al. (47). This is common among OUD patients who may encounter feelings of helplessness and sadness associated with the cycle of their substance use and periods of withdrawal. The people who use drugs and choose to seek treatment belong to a subset of the total population with OUD. They appear to experience more severe depressive symptoms, and consequently may be more compelled to seek help (56). In a multimodal treatment study in which all OUD patients received methadone, Rounsaville et al. (57) found that, despite the high number of subjects who complained of current or recent depressive symptoms, very few actually met criteria for an enduring MDD diagnosis. They hypothesized that this phenomenon may explain the apparent antidepressant effects of methadone for some patients: their depressive symptoms were transient and thus resolved by the treatment of their OUD. Simply entering OUD treatment may even resolve some depressive symptoms (58). Therefore, it is critical that OUD patients seeking treatment be screened for MDD prior to the initiation of opioid withdrawal, which may precipitate depressive symptoms but not clinical MDD.

### Randomized Controlled Trials

To our knowledge, the only randomized controlled trial comparing the antidepressant efficacy of buprenorphine and methadone was published by Dean et al. (46) in which they examined the first 12 weeks of a larger study conducted by NDARC comparing buprenorphine and methadone. Utilizing a double-blind design, 24 participants were randomized to the buprenorphine group and 30 patients were randomized to the methadone group. The average dose throughout the third month of treatment was 8.7 mg/day of buprenorphine or 47.9 mg/day of methadone. By the 3-month timepoint depressive symptoms had improved in all subjects and there was no significant difference in BDI between the buprenorphine and methadone groups. The average BDI at baseline was 24.7 for the buprenorphine group and fell to 13.4 at the 3-month timepoint. Similarly, the methadone group average BDI decreased from 22.2 to 11.6. The authors noted that higher baseline BDI scores predicted higher 3-month BDI scores in the methadone group only, suggesting that remission from MDD in OUD subjects with high baseline depression scores did not occur with methadone but may occur following buprenorphine treatment. While no discrepant antidepressant benefit of buprenorphine or methadone was found, future randomized controlled trials should expand on the differential predictive ability of baseline BDI scores. The full NDARC study included 200 buprenorphine-treated patients and 205 methadone-treated patients. Consistent with previous findings, there were no statistically significant differences in the efficacy of buprenorphine and methadone for the treatment of OUD as measured by urinalysis every 2 weeks and treatment retention at 3 months (50% vs. 59%, respectively) (59).

### Section Summary

The results of the studies reviewed here that evaluated whether methadone or buprenorphine is more efficacious for the treatment of MDD comorbid with OUD were inconclusive (Table 3). While two observational studies reported greater antidepressant effects for buprenorphine (54, 55), the only randomized controlled trial that compared this dimension of buprenorphine and methadone found no greater benefit for buprenorphine over methadone (46). Interestingly, the authors did note that higher BDI scores at baseline predicted higher BDI scores at the 3-month timepoint among the methadone group but not the buprenorphine group, suggesting that although the group average BDI scores were similar, buprenorphine treatment may have been more beneficial than methadone for the most severely depressed patients (46). This point corroborates data from Gerra et al. who found that patients with both OUD and MDD were more likely to achieve remission of depressive symptoms and more likely to abstain from opioid use when treated with buprenorphine vs. methadone.

**TABLE 3:** Antidepressant efficacy of buprenorphine vs. methadone. Outcomes of trials directly comparing the antidepressant efficacy of buprenorphine and methadone are mixed, with two finding that buprenorphine is superior and one finding no differential benefit between the medications.

References	Study design	Subjects	Dose: Mean (SD)	MDD rating scale	Baseline score: Mean (SD)	Final score: Mean (SD)	Outcome
(54)	Observational 3 months	76 buprenorphine-treated: 60 OUD only patients and 16 OUD + MDD patients	9.2 (3.4) mg/day	SCL-90 depression subscale	Not reported	Not reported	SCL-90 depression subscale scores decreased in both groups, but more so for the buprenorphine-treated group
		78 methadone-treated: 68 OUD only patients and 14 OUD + MDD patients	81.5 (36.4) mg/day	SCL-90 depression subscale	Not reported	Not reported	
(55)	Observational 9 months	Buprenorphine: 83 OUD patients	7.6 (4.6) mg/Day	SCL-90 depression subscale	.60 (.6)	.39 (.6)	SCL-90 depression subscale scores decreased in both groups, but more so for the buprenorphine-treated group
		Methadone: 80 OUD patients	69.4 (26.4) mg/day	SCL-90 depression subscale	.79 (.8)	.67 (.7)	
(46)	RCT 3 months	Buprenorphine: 24 OUD patients	8.7 (4.1) mg/day	BDI	24.7 (11.0)	13.4 (10.2)	Depressive symptoms among both groups improved, no between-group differences
		Methadone: 30 OUD patients	47.9 (20.1) mg/day	BDI	22.2 (10.5)	11.6 (10.4)	

MDD, major depressive disorder; OUD, opioid use disorder; RCT, randomized controlled trial; BDI, beck depression inventory; SCL-90, Symptom Checklist 90; SD, standard deviation.

Given that a substantial proportion of the OUD population seeking treatment has comorbid MDD, the antidepressant effect of a patient’s pharmacotherapy should be considered carefully before choosing buprenorphine or methadone. Although the limited data directly comparing the antidepressant benefits of buprenorphine and methadone are overall inconclusive, when coupled with outcomes from trials examining buprenorphine alone (Table 2) they suggest that buprenorphine may be preferentially beneficial for dually diagnosed patients.

An important consideration for interpretation of data comparing methadone and buprenorphine is differences in treatment access. Data generated from a secondary analysis of the 5-year Starting Treatment with Agonist Replacement Therapies (START) study (60) detailed OUD pharmacotherapy use after treatment tapering during a 32-week randomized trial of methadone or buprenorphine/naloxone. Out of 593 subjects with OUD, 85 had an MDD diagnosis. Relative to those with a history of OUD but no additional mental health disorder, individuals with comorbid OUD and MDD engaged with pharmacotherapy for a longer period of time during follow up (71.6% vs. 50.6%). Within that population, 60% of those using pharmacotherapy were using methadone (60). The authors highlighted the fact that this population was largely impoverished and had greater access to methadone treatment than buprenorphine. Two factors should address this lack of access: 1) Concerns regarding diversion of buprenorphine can largely be overcome by use of extended-release subcutaneous implants or depot injections that are now available and 2) training requirements to become a buprenorphine-waivered practitioner have been relaxed somewhat and improve access. This has allowed physicians, Nurse Practitioners, Physician Assistants, Clinical Nurse Specialists, Certified Registered Nurse Anesthetist, and Certified Nurse-Midwifes to avail of a Buprenorphine Waiver Notification of Intent (NOI) to treat up to 30 patients without the intensive training requirements. Another aspect of methadone treatment that could impact utilization of methadone over buprenorphine is the requirement for daily visits to the clinic. Even factors such as limited social support may impact the continuation of treatment long term. Despite the variability of the OUD patient population, continued examination of the antidepressant effects of buprenorphine compared to methadone in future clinical studies can identify differential predictors of efficacy. Going forward, it may also be useful to compare the efficacy of buprenorphine paired with behavioral therapy versus buprenorphine alone.

## Buprenorphine Can Treat Acute Suicidal Ideation in Opioid-Naïve and -Experienced Populations

Suicide is an ongoing and urgent threat to the millions of people in the United States suffering from mental health issues, especially MDD and OUD. In 2017, more than 47,000 people died by suicide in the United States (61). In 2018, 1.4 million individuals had a non-fatal suicide attempt (61) and 46,802 people died of an overdose involving opioids (62). The dual epidemics of suicide and opioid overdose are inextricably intertwined, as intentionality after an overdose death can be extremely difficult to discern in the absence of a suicide note. Taking into account opioid overdose deaths of “undetermined” intentionality, it is estimated that at least 20–30% of opioid overdose deaths are suicides (63). People with substance use disorders (SUDs) are six times more likely to attempt suicide than those without (64). Approximately 37% of heroin users entering the ATOS study had a lifetime suicide attempt and 14% had attempted suicide in the past year. In this cohort, a recent suicide attempt was associated with MDD (65), which was also a significant risk factor for suicidal ideation (66). Among the large STAR*D cohort, 53.3% of MDD patients with an SUD experienced suicidal ideation compared to 47.2% of MDD patients without an SUD (39). There is a compelling need to address suicidal ideation among both opioid-naïve and opioid-experienced populations.

### Case Report

Preliminary evidence from opioid-experienced subjects suggests that buprenorphine’s effects may be much stronger in the MDD and OUD population. In the first case report of buprenorphine’s ability to ameliorate suicidal ideation, Striebel and Kalapatapu (67) presented Ms. S, a 61 year-old woman with OUD, TRD, chronic pain, and suicidal ideation, among a host of other physical health conditions. After starting sublingual buprenorphine/naloxone treatment for her OUD at 16/4 mg/day, buprenorphine effectively managed her opioid craving and withdrawal symptoms, eliminated her depressive symptoms and suicidal ideation, and effectively treated her chronic pain. She remained on buprenorphine/naloxone treatment, with no suicidal ideation noted up to 3 months later. The case of Ms. S illustrates how the confluence of depression (with or without suicidal ideation), opioid misuse, and chronic pain can be particularly difficult to treat. Buprenorphine may offer the closest option to a one-size-fits-all approach by addressing all three concerns with one medication.

### Randomized Controlled Trials

There is evidence to suggest that low-dose buprenorphine may be an effective treatment for suicidal ideation in the opioid-naïve population (Table 4). Yovell et al. (68) established that with an average dose of just .44 mg/day, severely suicidal patients who received sublingual buprenorphine as an adjunctive therapy with their ongoing treatment regimen had a greater reduction in Beck Scale for Suicidal Ideation (BSSI) scores relative to patients who received placebo. The average BSSI scores at baseline in the buprenorphine and placebo groups were 19.7 and 19.6, respectively, demonstrating severe suicidal ideation. At the end of week two, the mean score change among the buprenorphine group was about −9, whereas the difference in the placebo group score was approximately −4.5. At the 4-week timepoint, the buprenorphine group average BSSI change was around -9.5 and the placebo group change was about −2. While the buprenorphine group BSSI score change was significantly greater than that of the placebo group at both timepoints, neither group score met the threshold for remission (69). This difference between groups held true using the Suicide Probability Scale (SPS) but was not significant on the BDI.

**TABLE 4:** Buprenorphine for the treatment of suicidal ideation. Buprenorphine, especially at moderate-to-high doses, is an effective treatment for suicidal ideation in both opioid-naïve and opioid-experienced populations.

References	Design	Subjects	Dose: mean (SD)	Suicidal ideation rating scale	Baseline score: mean (SD)	Final score: mean (SD)	Outcome
(67)	Case report	1 OUD + MDD patient with suicidal ideation	16 mg/day	Clinican assessment	N/A	N/A	Ms. S achieved remission from OUD and MDD symptoms and no longer had thoughts of suicide
(52)	RCT 3 days (single administration)	16 OUD + MDD patients	32 mg	BSSI	8.50 (8.53)	.625 (2.50)	All patients experienced amelioration of suicidal ideation at 3 days; maintained at 2-week follow-up
		17 OUD + MDD patients	64 mg	BSSI	11.05 (9.58)	1.17 (4.58)	
		14 OUD + MDD patients	96 mg	BSSI	8.24 (6.08)	.00 (.00)	
(68)	RCT 4 weeks	Buprenorphine-treated: 40 suicidal patients, (no OUD	.44 mg/day	BSSI	19.7	Not reported	Augmentation study; buprenorphine-treated patients had a greater mean score change than placebo-treated; average BSSI among both groups remained relatively high
		Placebo-treated: 22 suicidal patients, (no OUD)	0 mg/day	BSSI	19.6	Not reported	

MDD, major depressive disorder; OUD, opioid use disorder; RCT, randomized controlled trial; BSSI, beck scale for suicidal ideation; SD, standard deviation.

Using a design nearly identical to their randomized controlled trial of buprenorphine for OUD and MDD (51), Ahmadi et al. (52) demonstrated that a single dose of 32, 64, or 96 mg of sublingual buprenorphine can also treat suicidal ideation. The 47 male inpatients diagnosed with both OUD and MDD and reporting suicidal ideation were randomized into the three dosage groups and treated while experiencing moderate withdrawal symptoms. They found a significant reduction in BSSI across all three groups but no between-group differences: the 32 mg group average score fell from 8.50 at baseline to .625 at day three, the 64 mg group score from 11.05 to 1.17, and the 96 mg group score from 8.24 to .00. After 3 days of monitoring, no patients were experiencing suicidal ideation; this effect was maintained at the 2-week follow-up and no patients reported a “rebound” of suicidal ideation.

### Section Summary

It is essential to note that because the Yovell et al. (68) study recruited for suicidal ideation specifically, the research subject pool was very diverse and should not be generalized to suicidal ideation in the MDD population. The majority (56.8%) of patients enrolled met the criteria for borderline personality disorder (BPD). This is an important clinical population to examine in this context because personality disorders (PDs) are a risk factor for suicide (66) and the prevalence of PDs is significantly elevated among OUD patients. Strikingly, 72% of heroin-dependent entrants to the ATOS study met criteria for antisocial personality disorder (ASPD) and 47% met criteria for BPD (41), compared to estimated rates of 1–4% (70) and 1% (71) in the general population, respectively. While buprenorphine has demonstrated efficacy in a small group of opioid-naïve and opioid-dependent subjects with BPD specifically (72), retrospective analyses of Gerra et al.s’ 2004 paper revealed that buprenorphine was significantly less effective for OUD patients with comorbid antisocial or borderline PD (73). Therefore, while Yovell et al. (68) found no differences in response to buprenorphine between suicidal patients with BPD and suicidal patients without BPD, their results cannot necessarily be generalized to a more standard MDD population. Researchers investigating whether buprenorphine can effectively treat acute suicidal ideation in the future may want to screen for PDs and either exclude PD patients from the study or analyze them separately from MDD patients.

Regardless, these preliminary results suggest that buprenorphine is effective at treating suicidal ideation in both opioid-naïve and opioid-experienced populations. It is understood that MDD and personality disorders are both risk factors for suicide, but further research is required to disentangle their individual relationships to suicidal ideation and the pharmacotherapeutic utility of buprenorphine.

## Hypothesized Mechanism of Action for Buprenorphine’s Antidepressant Activity

### Preclinical Evidence

Substantial evidence has accumulated about the pharmacological effects of buprenorphine from preclinical studies to rationalize that buprenorphine may produce clinical effects as an antidepressant or to reduce suicide ideation. Buprenorphine is most characterized as a partial agonist at MORs because of investigations related to its ability to counteract the abuse potential of other opioids, like heroin or oxycodone. In addition, buprenorphine is a potent antagonist at KOR, and also antagonizes DOR and nociception/orphanin FQ (NOP) receptors at higher concentrations (11). The actions at KORs in particular provide a rationale for buprenorphine’s activity as an antidepressant drug and its ability counteract the effects of environmental stress.

Several preclinical studies have investigated the behavioral and neurochemical impact of buprenorphine in the context of stress, utilizing pharmacological tools to determine which of the opioid receptors mediates buprenorphine’s effects. Buprenorphine effectively normalized the social avoidance evoked in mice following exposure to a 10 days chronic social defeat stress paradigm (74) and reversed the behavioral deficits produced by 3 weeks of unpredictable chronic mild stress evaluated on the dark-light emergence, sucrose preference and forced swim tests (75). It was suggested that the ability of buprenorphine to reduce immobility scores in the mouse forced swim test (FST), a behavioral effect produced by all approved antidepressant medications, is strongly associated with its ability to block KORs. The effects of buprenorphine in this behavioral test were similar in magnitude to that produced by the selective kappa opioid receptor (KOR) antagonists nor-BNI and LY2456302 (also known as CERC-501, JNJ-6795396, and now Aticaprant) tested under similar conditions (74, 75). Buprenorphine failed to reduce immobility in mice with a genetic deletion of KORs (75). In contrast, mice with genetic deletion of MORs and DORs exhibited no alteration in buprenorphine reduced immobility. Furthermore, neither pretreatment with the NOP antagonist JTC-801 (75), nor co-administration with the opioid antagonist naltrexone (76) diminished buprenorphine’s behavioral activity in the FST. These studies support the idea that buprenorphine produces behavioral effects in the FST by KOR antagonism.

Curiously, on assays relevant to anxiety, it appears that the prolonged occupancy of MORs by buprenorphine, at a time when its agonist activity has waned, (24 h following a single injection) was responsible for mediating buprenorphine’s activity in the novelty induced hypophagia assay (77–79). At this time point buprenorphine exhibited functional antagonism at MORs. Based on these findings, it was hypothesized that both KOR and MOR antagonism contribute to an antidepressant/anxiolytic profile of buprenorphine. This beneficial pharmacological profile is supported by the comparable behavioral effects of the buprenorphine analog BU10119, an antagonist at both MOR and KOR (80). Also, nalmefene, a derivative of the pan-opioid antagonist naloxone with a 4-fold higher affinity for KORs relative to MORs produces similar behavioral effects across a range of behavioral assays without producing rewarding effects such as are evident in conditioned place preference paradigms (81). Nalmefene is approved for the treatment of alcohol use disorder in Europe.

At the neurochemical level, buprenorphine normalizes dopamine (DA) neurotransmission within the Nucleus accumbens (NAc), especially if it is disrupted by the effects of stress. In sophisticated microdialysis studies conducted in awake, behaving mice, mice trained to receive peanut butter chips showed an immediate 30% increase of extracellular DA release within NAc, but not in the striatum. Exposure to novelty stress (bright lights, odor) dramatically increased their latency to approach the food, and correspondingly prevented the increased DA release upon consumption of the peanut butter chips. Pretreatment with buprenorphine reduced approach latencies and restored the increase of DA release (82). These data stand as a figurative example supporting the hypothesis that buprenorphine mitigates the behavioral impairing effects of stress by modulating DA release in the NAc.

There is a substantial literature supporting a key role of KORs in behavioral stress, negative affect, and anhedonia, including the effects of drug withdrawal [for review see (83, 84)]. Most of these data were obtained in healthy animals, or in healthy animals exposed to stress paradigms. It is possible that buprenorphine’s antidepressant activity in individuals with OUD may occur through differential neural mechanisms than those required in subjects with MDD alone and this has yet to be investigated using appropriate models. The involvement of KORs in negative affect provides a framework for the investigation of translational signals in humans. For example, long term opioid exposure increases levels of the endogenous ligand for KORs, dynorphin, resulting in greater dysphoria and drug seeking in animals (84). In humans, peripheral blood lymphocyte KOR mRNA expression was robustly decreased in individuals with an OUD, those receiving methadone maintenance therapy and in those that were abstinent for at least 12 months (85), suggesting that decreased levels of KOR is a trait marker of prolonged opioid exposure. In contrast, a similar assessment of peripheral KORs in individuals with MDD, reported an increase in KOR protein during a current episode of depression (86). These data are striking but the methods used and results have yet to be corroborated in other laboratories. Nevertheless, these findings support targeting dynorphin/KOR signaling abnormalities to attenuate the dysphoric and negative affective state associated with substance use disorders in general. Therefore, investigation of buprenorphine’s ability to normalize peripheral KOR expression in both MDD and OUD alone and together is warranted given its antagonist activity at KORs.

### Section Summary

There is a substantial body of preclinical literature suggesting that buprenorphine’s activity at KORs mediate its antistress and antidepressant activity. However, there remains little information regarding buprenorphine’s ability to normalize aberrant dynorphin/KOR signaling in the context of OUD and MDD and more extensive evaluation of the neurochemical correlates of this are required in future studies.

## Future Directions

This review has pointed to several gaps in the current literature on buprenorphine that must be filled by further research. The most pressing challenge is to establish dose-response curves for buprenorphine in the opioid-naïve and opioid-experienced populations. Karp et al. postulated that long-term treatment with low doses of buprenorphine may be required to sustain antidepressant effects in opioid-naïve patients (21). In contrast, evidence from patients with OUD and MDD who received higher doses of buprenorphine repeatedly suggest that there may be a ceiling effect to buprenorphine’s antidepressant action (46, 51, 52). This was contradicted by Ahmadi et al., that who demonstrated that the antidepressant effects of a single high dose of buprenorphine were maintained 2 weeks later (51, 52). Perhaps increasing doses of buprenorphine does not increase the magnitude of the antidepressant effect, but the duration of action. Future studies would be strengthened by the addition of long-term follow-up assessment.

Another issue in the field of comorbid MDD and OUD is inconsistent or inadequate symptom assessments related to depression. Parsing apart the ability of buprenorphine and other treatments to ameliorate clinical symptoms of MDD from their ability to address transient OUD-related depressive symptoms is an important requirement for OUD treatment studies. Baseline measurements of mood should begin before the patient has gone into opioid withdrawal and be continued throughout the study and use adequate assessment instruments. A comprehensive mental health history can help address whether the temporal relationship of depression onset and the initiation of substance use is relevant to treatment efficacy (48). For longer study periods, symptom surveys administered at least biweekly would allow for closer therapeutic time course tracking. Furthermore, utilizing consistent OUD and MDD rating scales (e.g., ASI and BDI) would facilitate cross-study comparison and increase replicability.

Finally, the population that buprenorphine has been tested in is exceedingly heterogeneous. It may be beneficial to narrow the scope of some future studies by focusing on patients with OUD alone, MDD alone, or MDD and OUD without other comorbid disorders. Given the high prevalence of psychiatric comorbidity and polydrug use, compounded with high dropout rates in this population, this can be a challenging task. Pharmacogenomics is an emerging tool that could be useful for standardizing subject pools and personalizing treatment regimens. There is an indication that the presence of certain alleles can influence optimal dosage and impact the overall efficacy of buprenorphine for a given individual (87).

Whether an OUD patient primarily uses heroin or prescription opioids may also influence the dosage and efficacy of buprenorphine. The dose of buprenorphine necessary to manage OUD symptoms and achieve an antidepressant effect may be lower for prescription opioid users than heroin users (45). Additionally, current prescription opioid users in the POATS study with any lifetime heroin use were half as likely to have a favorable outcome on buprenorphine treatment (48). Information about which drugs OUD patients are using and their history should be collected and considered throughout analysis when possible.

Overall, the set of studies reviewed here support buprenorphine as a promising therapeutic candidate for MDD and OUD, for which further research is certainly warranted. Addressing many of these methodological considerations when designing future studies will strengthen this body of literature and allow clinical treatment to be delivered more effectively.

## Conclusion

Taken together, the studies discussed in this review tell a cohesive story: buprenorphine is an effective and safe antidepressant. Buprenorphine provides a unique antidepressant mechanism—opioid system modulation—which is a novel treatment option when used at low doses for many opioid-naïve individuals with TRD. At higher doses, buprenorphine can fulfill the dual role of staving off OUD symptoms and addressing MDD and pain which, given the high rate of comorbid MDD and OUD, are appealing properties for a therapeutic. Furthermore, buprenorphine has had success in diminishing suicidal ideation in both opioid-naïve and opioid-experienced groups. Although the abuse potential of buprenorphine is considered low, new forms of buprenorphine involving depot injections or implants have been introduced recently that could counteract lingering concerns about this potential liability. Finally, despite conflicting results in trials comparing the antidepressant effects of buprenorphine and methadone, it is clear that buprenorphine is as effective as methadone at managing OUD. Considering the robust antidepressant effects in patients with OUD and MDD reported elsewhere, it seems reasonable to favor buprenorphine for dually diagnosed patients.

Many of the studies discussed in this review were limited by issues such as small sample sizes, heterogeneous subject pools, and/or a lack of control arm. Nevertheless, the overall literature indicates that further research into the utility of buprenorphine as an antidepressant is warranted. Future research must build on the existing literature with high-powered randomized controlled trials that explicitly include depressive symptomology as an outcome measure.
